# Peer Review of “Forecasting the COVID-19 Pandemic in Saudi Arabia Using a Modified Singular Spectrum Analysis Approach: Model Development and Data Analysis”

**DOI:** 10.2196/28741

**Published:** 2021-03-31

**Authors:** Anonymous Reviewer

**Keywords:** COVID-19, prediction, singular spectrum analysis, separability, eigenvalues, Saudi Arabia

*This is a peer review submitted for the paper "Forecasting the COVID-19 Pandemic in Saudi Arabia Using a Modified Singular Spectrum Analysis Approach: Model Development and Data Analysis".*

## Round 1 Review

The paper is very interesting and very timely. I would suggest publishing it after minor revision.

Some information on the singular spectrum analysis technique could be added. Some additional information on COVID-19 cases could also be provided.

When one model outperforms another, it should be statistically tested. Considering the explanation of various parts as well as forecasting error, it is advisable to use a test that does not depend on the normality of error as well as h-step ahead forecasting.
